# Versatile artificial *mer* operons in *Escherichia coli* towards whole cell biosensing and adsorption of mercury

**DOI:** 10.1371/journal.pone.0252190

**Published:** 2021-05-26

**Authors:** Nai-xing Zhang, Yan Guo, Hui Li, Xue-Qin Yang, Chao-xian Gao, Chang-ye Hui

**Affiliations:** 1 National Key Clinical Specialty of Occupational Diseases, Shenzhen Prevention and Treatment Center for Occupational Diseases, Shenzhen, China; 2 Department of Pathology & Toxicology, Shenzhen Prevention and Treatment Center for Occupational Diseases, Shenzhen, China; University of Houston, UNITED STATES

## Abstract

Mercury exists naturally and mainly as a man-made pollutant in the environment, where it exerts adverse effects on local ecosystems and living organisms. It is important to develop an appropriate synthetic biological device that recognizes, detects and removes the bioavailable fraction of environmental mercury. Both single-signal and double-signal output mercury biosensors were assembled using a natural *mer* operon as a template. Selectivity and sensitivity of whole-cell biosensors based on artificial *mer* operons were determined. Three whole-cell biosensors were highly stable at very high concentrations of mercuric chloride, and could detect bioavailable Hg(II) in the concentration range of 6.25–200 μM HgCl_2_. A novel Hg(II) bioadsorption coupled with biosensing artificial *mer* operon was assembled. This would allow Hg(II)-induced Hg(II) binding protein cell surface display and green fluorescence emission to be achieved simultaneously while retaining the linear relationship between fluorescent signal and Hg(II) exposure concentration. The present study provides an innovative way to simultaneously detect, quantify, and remove bioavailable heavy metal ions using an artificially reconstructed heavy metal resistance operon.

## Introduction

Mercury is a bioaccumulative and highly toxic heavy metal that is widely dispersed in the environment. Environmental mercury exists in three different forms: elemental mercury, inorganic mercury, and organic mercury. Among these forms, organic methyl mercury poses a significant hazard to public health and safety [1]. Although there are a substantial number of instrumental methods available for the determination and quantification of mercury in different environmental samples, there is a lack of information in speciation studies of mercury in recent years [2]. Measurement of bioavailable Hg(II) has predictive value for the methylation rate of mercury, thereby predicting its biological accumulation in ecosystems [3]. Thus, it is imperative to develop appropriate biological devices which detect and remove the bioavailable Hg(II) in the environment.

Due to environmentally widespread toxicity of mercury, it has been evolutionarily necessary for bacteria to evolve resistance to mercury. Bacteria surviving in heavy-metal polluted environments rely on the function of specific heavy-metal resistance systems. One of the best understood microbial mercury resistance operons is the *mer* operon that confers microbial resistance to inorganic mercury [4, 5]. Bacterial metalloregulatory MerR is a Hg(II) dependent transcriptional repressor and activator that responds to Hg(II) with high selectivity and sensitivity. Apo MerR dimer binds to the promoter region of the *mer* operon as a repressor to block transcription initiation of a downstream mercury detoxification gene cluster. However, this dimeric MerR is converted into an activator upon Hg(II) binding [6]. Several whole-cell biosensors to detect bioavailable Hg(II) were successfully developed using the Hg(II) response elements originating from the natural *mer* operon. These single-signal output biosensor constructs responded to bioavailable Hg(II) by producing light, β-galactosidase, fluorescent protein, or pigment [7–10]. Due to its high affinity and selectivity toward Hg(II), MerR has been genetically engineered onto the surface of bacteria to develop microbial biosorbents specific for Hg(II) removal [11, 12]. These findings show that biological engineering of the mercury resistance operons of natural origin could provide an alternative way for the control of mercury pollution.

So far, a few approaches have been used to assemble a single-signal biosensor to detect and quantify bioavailable Hg(II). However, multiple-signal output biosensors have been demonstrated to provide more information and more flexible detection methods than traditional single-signal output biosensors [13–16]. Furthermore, integration of biosensing and biosorption can be realized using a multiple-signal output genetic device as a template [16–18]. In this study, based on a natural *mer* operon originating from the *E*. *coli* transposon Tn21 [19], both artificial dicistronic *mer* operon and artificial double-promoter *mer* operon were designed, constructed, and validated for double-signal biosensing of mercury. The responses of all the biosensors to Hg(II) were quantitative. Simultaneous biodetection and bioremediation of Hg(II) were finally achieved using a double-promoter regulated artificial *mer* operon. Overall, this study provides an example of how to assemble artificial heavy metal response systems using natural metal resistance operons as templates for biological detection and recovery of bioavailable heavy metals.

## Materials and methods

### Bacterial strains, plasmids, and agents

The bacterial strain and vectors involved in this study are listed in Table 1. *E*. *coli* TOP10 was used as a host strain for both cloning and expression of recombinant proteins. Cultures were grown in Luria Broth (OXOID, Basingstoke, UK) supplemented as necessary with ampicillin at a final concentration of 50 μg/mL. Purification of PCR products and plasmids were performed with kits from Sangon Biotech (Shanghai, China). All chemicals were purchased from Sigma-Aldrich (St Louis, MO, USA). Stock solutions of CdCl_2_, CaCl_2_, MgCl_2_, FeSO_4_, MnSO_4_, NiSO_4_, CuSO_4_, ZnSO_4_, Pb(NO_3_)_2_, and HgCl_2_ were freshly prepared with analytical grade chemicals and distilled water. DNA synthesis and sequence verification of all constructed vectors were performed by Sangon Biotech.

**Table 1:** Bacterial strain, plasmids, and primers used in this study.

Strain and vectors	Genotypes or description	Reference
Strain		
*E*. *coli* TOP10	F^-^, Φ80*lac*ZΔM15, Δ*lac*X74, *rec*A1	Invitrogen
Plasmid		
pET-21a	Amp^R^, T7 promoter, lac operator	Novagen
pT-RFP	T vector carrying *mcherry*	[20]
pT-GFP	T vector carrying *egfp*	[20]
pPmer	pET-21a derivative containing *merR* and Pmer divergent promoter region cloned into *Bgl*II and *Xba*I sites	This study
pPmer-R	pPmer derivative carrying promoterless *mcherry* cloned into *Nde*I and *Hin*dIII sites	This study
pPmer-R-G	pPmer derivative, an artificial two-cistron *mer* operon with a translationally coupled *mcherry* and *egfp* cassette	This study
pPmer-R-Pmer-G	pPmer derivative, an artificial hybrid *mer* operon with transcriptions of *mcherry* and *egfp* under the control of independent Pmer divergent promoter region	This study
pPmer-LOA-Pmer-G	pPmer derivative, an artificial hybrid *mer* operon with transcriptions of *lpp-ompA* and *egfp* under the control of independent Pmer divergent promoter region	This study
pPmer-HgBD-Pmer-G	pPmer derivative, an artificial hybrid *mer* operon with transcriptions of *lpp-ompA-HgBD* and *egfp* under the control of independent Pmer divergent promoter region	This study

### Cloning and construct assembly

**Fig 1:**
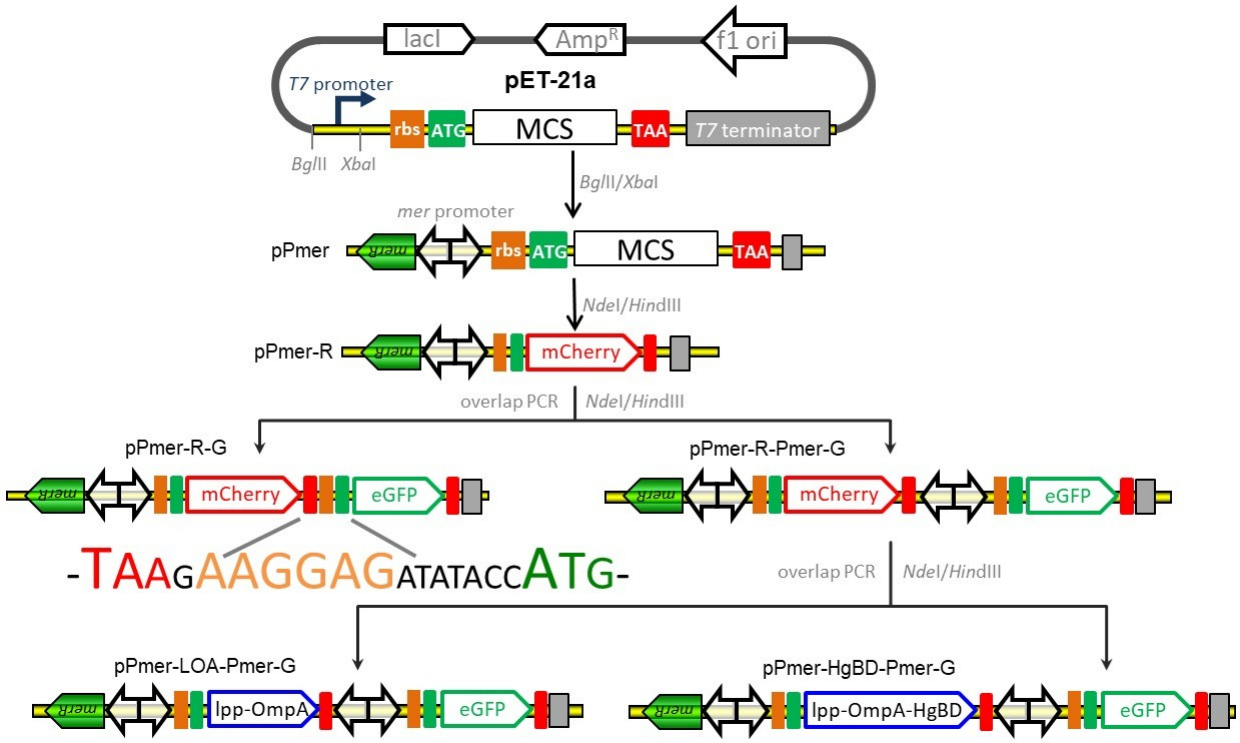
Assembly of artificial *mer* operons for biosensing and adsorption of Hg(II). The fluorescent reporter modules and bioadsorption module were placed under the control of the *mer* promoter separately or in combination. The DNA sequence containing the stop codon of the upstream gene, an extra RBS, and the start codon of the downstream gene is shown.

The strategy used for the assembly of the constructs for mercury biosensing and adsorption is summarized in Fig 1. The DNA sequence of the expression and regulation regions of recombinant plasmids involved in the study are shown in S1 Fig. The DNA fragment coding for Hg(II)-responsive metalloregulatory protein MerR and the divergent operator-promoter region (NCBI Accession No. AF071413.3) was synthesized, and introduced into the *Bgl*II/*Xba*I cloning sites of the plasmid pET-21a to generate pPmer. In order to construct a single-signal output mercury biosensor, a promoterless *mcherry* gene was PCR amplified from the vector pT-RFP, and inserted into the *Nde*I and *Hin*dIII sites of pPmer to generate pPmer-R. Two strategies were then used for the assembly of double-signal output biosensors. Firstly, a DNA module containing the ribosome binding site (RBS) and the eGFP-encoding sequence was PCR amplified from pT-GFP, and fused with the mCherry-encoding sequence in pPmer-R by an overlapping extension PCR as described previously [16] to generate pPmer-R-G, which was designed as an artificial dicistronic *mer* operon. Secondly, a DNA module containing an extra *mer* operator-promoter region and the eGFP-encoding sequence was PCR amplified from pT-GFP, and fused with the mCherry-encoding sequence in pPmer-R by an overlapping extension PCR to generate pPmer-R-Pmer-G, which was designed as an artificial double-promoter *mer* operon.

Hg(II) binding domain (HgBD) derived from MerR was fused with a surface anchor system Lpp-OmpA to generate the chimera protein Lpp-OmpA-HgBD. The synthetic DNA fragments encoding Lpp-OmpA and Lpp-OmpA-HgBD were cloned into pPmer-R-Pmer-G for substituting the mCherry-encoding sequence with an overlapping extension PCR, to generate pPmer-LOA-Pmer-G and pPmer-HgBD-Pmer-G, respectively. The vector pPmer-HgBD-Pmer-G was designed as a Hg(II) inducible mercury adsorptive and biosensing construct.

### Specificity test

Single-signal output biosensor *E*. *coli* TOP10/pPmer-R, double-signal output biosensors TOP10/pPmer-R-G and TOP10/pPmer-R-pPmer-G were activated overnight in LB medium. A total of 30 μL of each culture was inoculated into 3 mL of fresh LB medium, and the cells were grown at 37°C until OD_600_ = 0.4. Then a final concentration of 10 μM Cd(II), Ca(II), Mg(II), Fe(II), Mn(II), Ni(II), Cu(II), Zn(II), Pb(II), or Hg(II) was added to the medium, followed by culturing at 37°C for 12 h before assessment of reporter signals.

### Sensitivity test

Three recombinant biosensor strains were grown at 37°C overnight and inoculated into 3 mL of fresh LB medium at 1% inoculum. The cells were grown at 37°C until OD_600_ = 0.4. They were then induced by 0, 3.125, 6.25, 12.5, 25, 50, 100, 200, and 400 μM HgCl_2_ with shaking at 37°C for 12 h before assessment of reporter signals.

### Measurements of fluorescent signals

The fluorescent proteins generated from the engineered bacterial strains were quantitated with a Lumina fluorescence spectrometer (Thermo, USA) as previously described [20]. The excitation wavelength was set at 587 nm, and the emission wavelength was set at 610 nm for the reporter mCherry. The excitation wavelength was set at 488 nm, and the emission wavelength was set at 507 nm for the reporter eGFP. Then, the fluorescence intensity value was divided by the absorbance at 600 nm in order to normalize to bacterial cell concentration. The induced engineered bacterial cells were also visualized using a Nikon Eclipse Ni fluorescence microscope (Tokyo, Japan) as described previously [20]. The imaging reporter mCherry was visualized with a Texas Red filter, and the imaging reporter eGFP was visualized with a FITC filter.

### Evaluation of simultaneous Hg(II) bioadsorption and biosensing by the engineered bacteria

For the simultaneous detection and adsorption of Hg(II), two kinds of recombinant *E*. *coli* TOP10 harboring artificial *mer* operons pPmer-HgBD-Pmer-G and pPmer-LOA-Pmer-G (as the control) were grown at 37°C overnight, and inoculated into fresh LB medium at 1% inoculum. The cells were grown at 37°C until OD_600_ = 0.4, and then a final concentration of 0, 6.25, 12.5, 25, 50, 100, 200, and 400 μM HgCl_2_ were added to the medium, followed by culturing at 37°C for 12 h before measurement of the fluorescent signal and mercury binding capacities. For mercury analysis, the induced cells were washed extensively with saline, dried, and digested with the nitric acid. The cell-associated Hg(II) was finally determined using atomic absorption spectrometry as described previously [11, 21].

## Results and discussion

### Design of artificial *mer* operons

**Fig 2:**
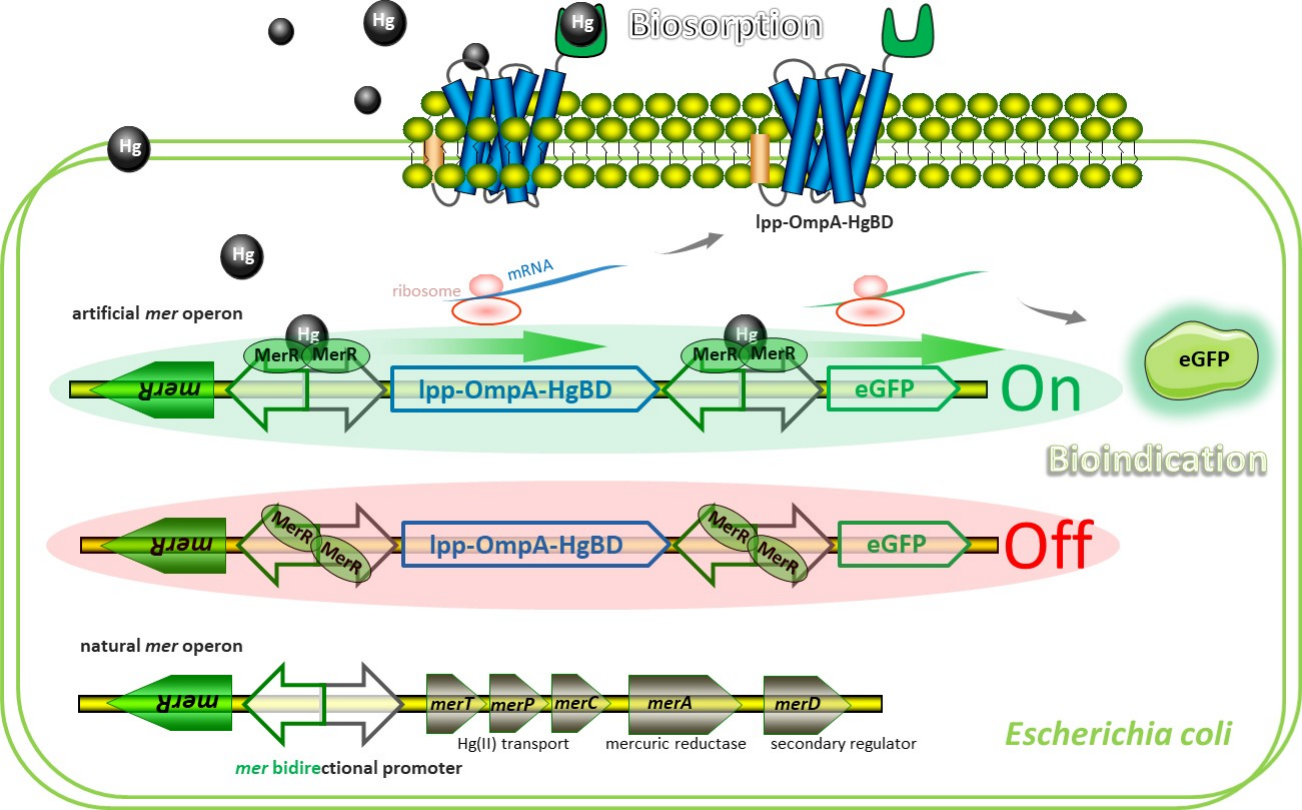
Models for natural and artificial *mer* operons. Model for artificial *mer* operon-encoded biosorption and bioindication of Hg(II). The model involves the following proteins: Lpp-OmpA-HgBD, an outer membrane anchoring chimera protein for surface display of metal binding domain derived from MerR; eGFP, a fluorescent reporter. Dimeric MerR bound to the *mer* divergent promoter activates transcription of the surface-display module and the reporter module in the presence of Hg(II) (top), and represses transcription in the absence of Hg(II) (middle). The archetype of natural *mer* operon is located on transposon Tn21 from *E*. *coli* (bottom). Binding of Hg(II) to dimeric MerR is demonstrated to result in DNA distortion and transcriptional activation of the downstream Hg(II) transport and detoxification genes.

The *mer* operon derived from the *E*. *coli* transposon Tn21 is the-best characterized mercury resistance system [19]. Natural *mer* operon is composed of a metalloregulator gene and a mercury detoxificated gene cluster, which are divergently transcribed under the control of *mer* promoter (S2 Fig). The sensing element is the MerR dimer, which represses transcription of *merTPCAD* in the absence of Hg(II) but activates transcription of *merTPCAD* in the presence of Hg(II). The merTPCAD genetic cassette was substituted with a mCherry gene cassette, a dicistronic mCherry-eGFP genetic cassette, and a double-promoter mCherry-Pmer-eGFP genetic cassette, to assemble a single-signal output biosensor construct pPmer-R, double-signal output biosensor constructs pPmer-R-G, and pPmer-R-Pmer-G, respectively. More importantly, a double functional element integrating bioadsorption and biosensing modules can substitute the *merTPCAD* gene cassette (Fig 2). It allows different functional elements to be transcribed under the control of its own promoter, followed by the surface display of HgBD for Hg(II) bioadsorption and the expression of eGFP for Hg(II) biosensing at the mean time.

### Mercury selectivity detection with single- and double-fluorescent signal biosensors

**Fig 3:**
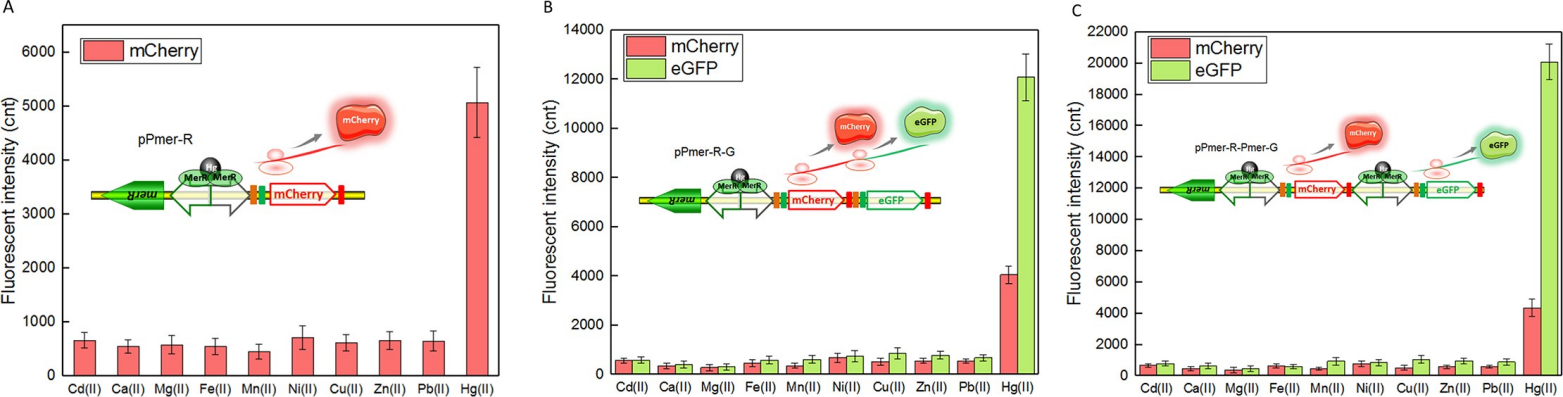
The response of three mercury biosensors exposed to different metal ions at 10 μM. After induction with 10 μM various metal ions at 37°C for 12 h, two kinds of fluorescent signals were all determined. The data were obtained by subtracting the value of recombinant *E*. *coli* with no metal ion exposure from that of each group. (A) TOP10/pPmer-R. (B) TOP10/pPmer-R-G. (C) TOP10/pPmer-R-Pmer-G. Fluorescence intensity values were divided by the absorbance at 600 nm in order to normalize to bacterial cell concentration. Data are representative of at least three independent experiments, and the values are expressed as mean ± SD.

It has been previously proven that the metalloprotein MerR is the Hg(II)-specific transcriptional regulator [4]. The traditional single-signal sensors employing MerR-like metalloproteins as sensing elements all showed extraordinary selectivity toward Hg(II) [7, 8, 10]. To study the effect of double-signal output genetic combination on the specificity of whole-cell biosensors, three engineered bacterial biosensors in logarithm growth period were exposed to different kinds of metal ions at 10 μM. As shown in Fig 3, all three whole-cell biosensors responded silently to all metal ions other than Hg(II). It was similar with the performance of single-signal biosensor (Fig 3A) that both artificial dicistronic *mer* operon (Fig 3B) and artificial double-promoter *mer* operon (Fig 3C) showed a selective response to Hg(II). The response strength of mCherry in single-signal biosensor was slightly higher than that in double-signal biosensors. Compared with the single-signal output construct, expression of an extra reporter eGFP increases energy and nutrient consumption. Thus decreased signal strength is expected in the double-signal output constructs [13, 16, 20]. Furthermore, the response strength of eGFP in the mode of double-promoter pattern (20090 cnt) was significantly higher than that of eGFP in the mode of dicistronic pattern (12078 cnt). It is well known that the processes of transcription and translation are coupled in bacteria. The expression of reporter is affected by many factors, including secondary structure of the mRNA, strength of the promoter, the efficiency of RBS, and more [22, 23]. Higher expression of the second reporter eGFP will be expected when transcription and translation are regulated under its own promoter.

### Mercury sensitivity detection with single- and double-fluorescent signal biosensors

**Fig 4:**
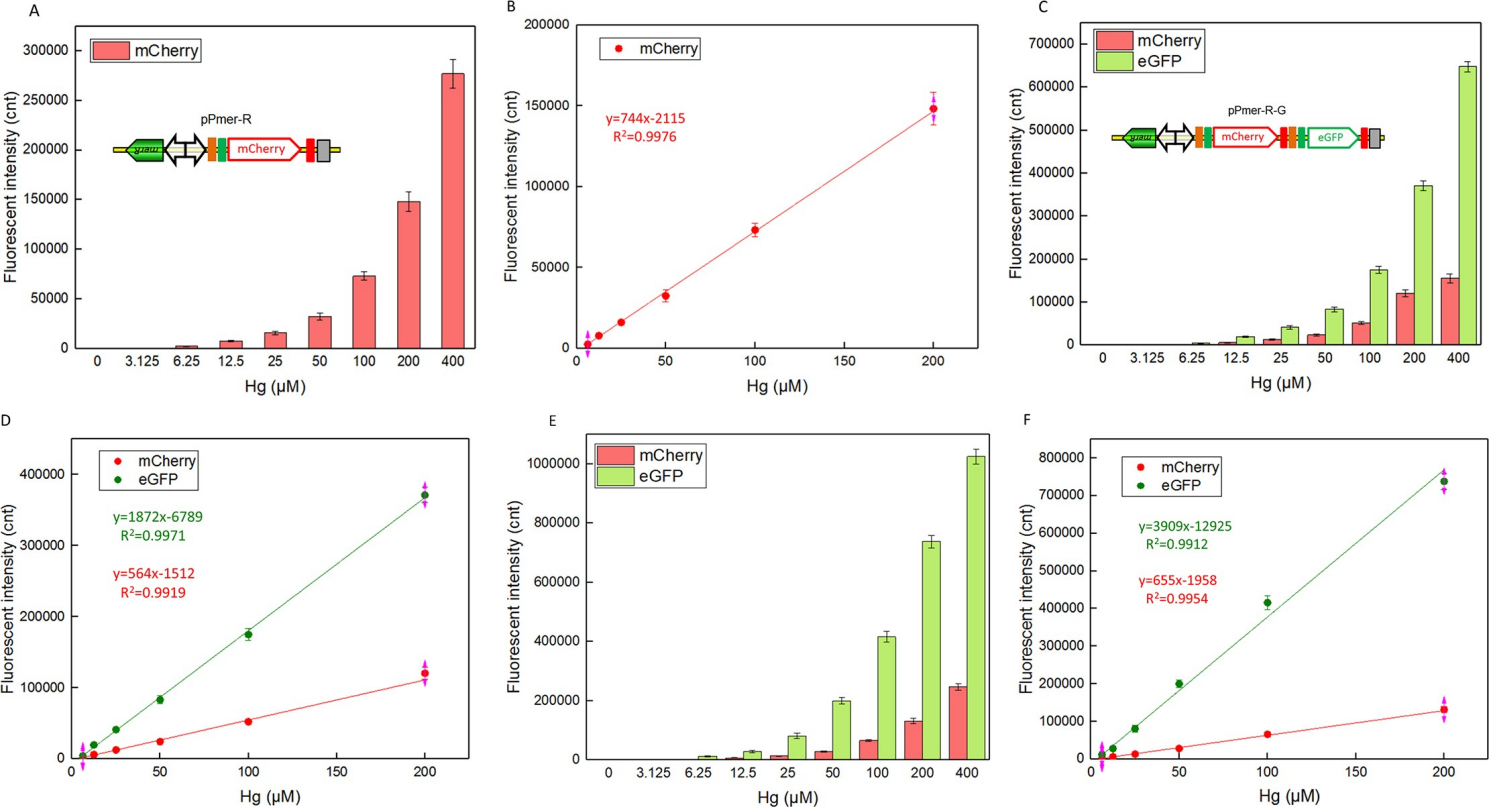
Comparison of reporter signals generated by three mercury biosensors after exposure to gradient concentrations of Hg(II). After induction with 0–400 μM Hg(II) at 37°C for 12 h, two kinds of fluorescent signals (mCherry and eGFP) were all determined. Whole-cell biosensor TOP10/pPmer-R dose-response (A) and linear response (B) to Hg(II), whole-cell biosensor TOP10/pPmer-R-G dose-response (C) and linear response (D) to Hg(II), and whole-cell biosensor TOP10/pPmer-R-Pmer-G dose-response (E) and linear response (F) to Hg(II). Fluorescence intensity values were normalized using the absorbance at 600 nm. The data were obtained by subtracting the value of recombinant *E*. *coli* with 0 μM Hg(II) exposure from that of each group. Values represent the mean ± SD of at least three independent experiments.

Three recombinant biosensor strains were then examined to determine their dynamic ranges of fluorescent responses to different concentrations of Hg(II). As shown in Fig 4, for all three engineered biosensors, the fluorescent signals increased with the concentration of Hg(II) in medium in the concentration range of 3.125–400 μM. It has been reported that a pigment-based engineered *Pseudomonas aeruginosa* PAO1 showed a good linearity for Hg(II) in the range of 25–1000 nM [10]. The GFP fluorescence emission showed a linear increase from 100 to 1700 nM Hg(II) in an *E*. *coli* biosensor [8]. A linear positive correlation was observed between 50 nM to 10 μM Hg(II) in an engineered *E*. *coli* with constitutively expressed MerR as a sensor protein and inducible mCherry as the reporter [9]. However, no whole-cell biosensors have been developed to be used in the upper ranges of Hg(II) concentrations where linearity was demonstrated currently. Based on a series of artificial *mer* operons, linear relationships between fluorescent signals and the concentration of Hg(II) were observed in both single-signal and double-signal output biosensors within the concentration range of 6.25–200 μM. Although the linear response range of these three biosensors is significantly higher than previously reported whole-cell biosensors, they tolerate the toxicity of high concentrations of Hg(II), and can be expected to be used in the quantification of the high concentration range of Hg(II) existing in heavily polluted environmental water samples.

The order of fluorescent response strength of mCherry in these three biosensors is TOP10/pPmer-R > TOP10/pPmer-R-Pmer-G > TOP10/pPmer-R-G. Compared with the single-signal output biosensor TOP10/pPmer-R (Fig 4A), the mCherry fluorescence decreased about 10% in artificial double-promoter biosensor TOP10/pPmer-R-Pmer-G (Fig 4E), and about 30% in artificial dicistronic biosensor TOP10/pPmer-R-G (Fig 4C), respectively. As expected, the response strength of eGFP derived from artificial double-promoter biosensor TOP10/pPmer-R-Pmer-G (Fig 4E) was significantly higher than that derived from artificial dicistronic biosensor TOP10/pPmer-R-G (Fig 4C). As a result, the following integration of Hg(II) bioadsorption genetic element and biosensing genetic element was done using an artificial double-promoter regulated *mer* operon as a template.

Resistance to inorganic mercury compounds is widely found among various eubacteria. The mercury resistance locus can occur on plasmids or on the genome, and confers resistance by reduction of Hg(II) to the volatile, less toxic elemental mercury [5, 19]. The exponential phase of the host TOP10 and three whole-cell biosensors could tolerate high concentration of Hg(II), while the growth of bacterial cells declined with Hg(II) exposure (S3 Fig). Although the bacterial density was not increased above 200 μM Hg(II) exposure, the enhanced fluorescent signals were still recorded (Fig 4). Compared with the single-signal output biosensor, double-signal output just exerted a slightly adverse effect on the growth of biosensor cells (S3 Fig). Time-response curves of three whole-cell biosensors toward 200 μM Hg(II) were shown in S4 Fig. Both of the fluorescent signals were increased with the extension of induction time. After an 8-h induction, the fluorescent signals were not enhanced any more. An overnight induction was chosen to facilitate the experimental arrangement in this study. Interestingly, an 8-h induction time was enough to obtain the maximum signal output.

A few highly sensitive Hg(II)-specific biosensors based on oligonucleotides or DNA-protein interactions have been reported [24–27]. As whole-cell biosensors are sensitive to only intracellular Hg(II), they are usually used to study the effects of mercury speciation on the bioavailability to the organisms and the maintenance of homeostasis [7, 28]. Based on the reconstruction of diverse microbial mechanisms that are responsible for maintenance homeostasis and resistance to mercury, some whole-cell biosensors that responded to mercury by producing fluorescent protein, β-galactosidase, pigment, and luminescence have been successfully developed [7, 10, 29, 30]. The luminescence biosensor with the highest sensitivity could detect concentrations as low as 0.02 μM Hg(II) after a 1.5-h induction [7, 29, 31]. To enhance the stability of whole-cell biosensors, the biosensing genetic element was integrated stably into the chromosome of the host, and the resultant biosensor could detect Hg(II) less than 0.2 μM after a 12-h induction [8, 32]. Novel visual reporters such as pigments have been used to develop whole-cell biosensors for enhanced sensitivity and stability [33–35]. The detection limit of whole-cell biosensor with pyocyanin as a signal output could reach as low as 0.01 μM Hg(II) after an overnight incubation [10]. In addition to the effects of reporters, optimization of detection conditions including the type of culture mediums, induction time and induction duration, was another important factor for enhanced sensitivity [13, 16, 36]. Although whole-cell biosensors responded to low concentration of heavy metal when lag-phase culture of biosensor cells was directly exposed to toxic metal [7, 10], exponential phase biosensor cells could produce significantly stronger reporter signals including fluorescent protein, β-galactosidase, and pigment [13, 35, 37, 38]. Furthermore, logarithmic phase bacterial cultures were usually induced to improve the expression amount of surface-displayed metal binding proteins [16, 18, 21, 37, 39]. Based on the above-mentioned factors, logarithmic phase biosensor cultures were chosen for high tolerance to toxic Hg(II) and high expression of surface-displayed HgBD in this study.

### Double-color fluorescent detection of whole-cell biosensors

**Fig 5:**
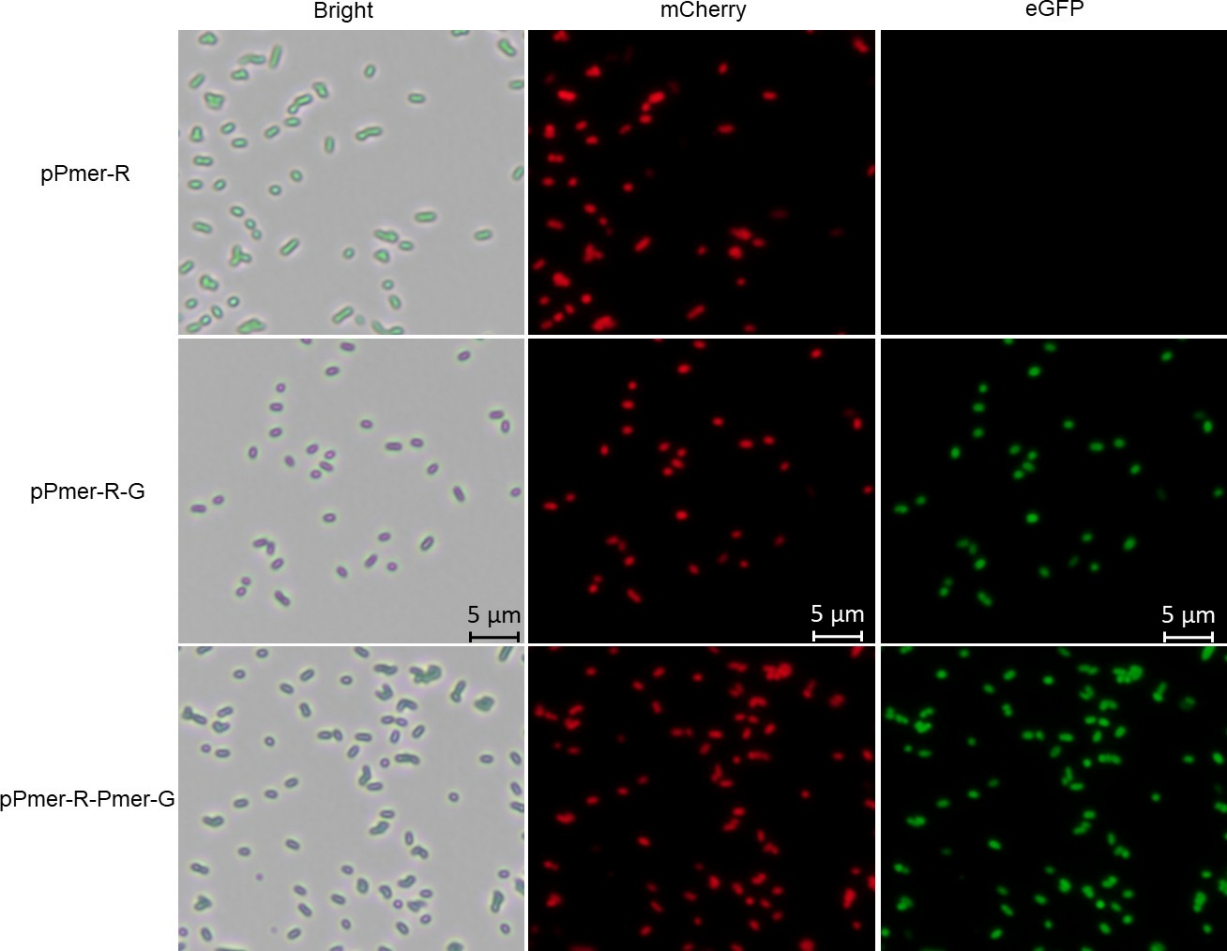
Fluorescence images of three mercury biosensors exposed to 400 μM Hg(II) at 37°C for 12 h. *E*. *coli* cells were visualized using a fluorescence microscope (×600 magnification). The mCherry expression was visualized with a Texas Red filter, and the eGFP expression was visualized with a FITC filter. Three independent preparations were analyzed, and representative images are shown here.

The performance of single biosensor cells could be conveniently assessed by fluorescent image using fluorescent reporter systems. The single-signal output biosensor TOP10/pPmer-R emitted red fluorescence after Hg(II) induction, which was visualized under a fluorescence microscope. The double-signal output biosensors TOP10/pPmer-R-G and TOP10/pPmer-R-Pmer-G emitted both red and green fluorescence (Fig 5). Multiple-signals output can provide more insights on detection and quantification of heavy metal than traditional single-signal biosensors can, especially when significant overlapping background fluorescence exists [40]. Double-color fluorescent signals also facilitate the analysis of biosensing signals based on flow cytometry [8, 38].

### Integrated mercury bioadsorption and biosensing based on an artificial double-promoter *mer* operon

**Fig 6:**
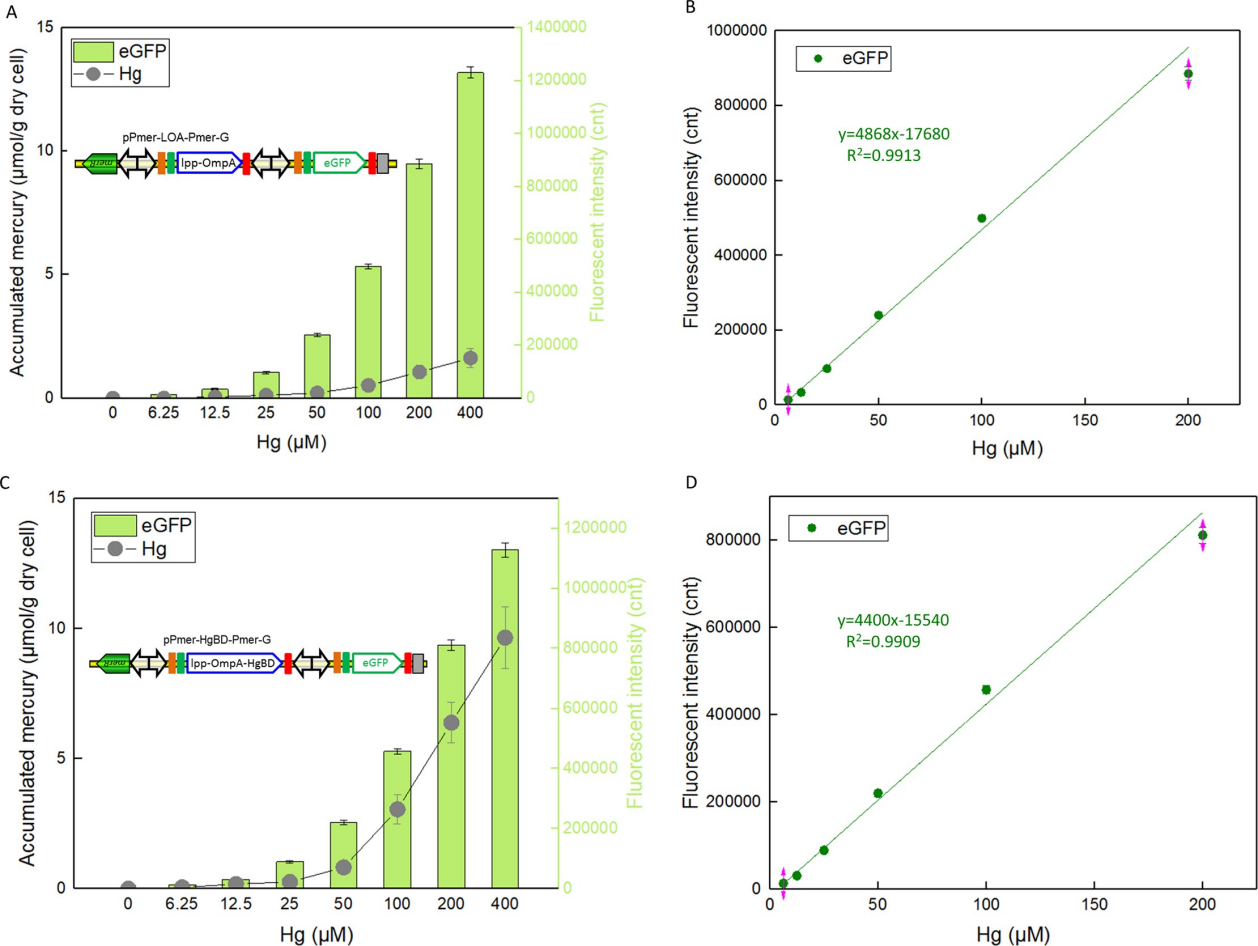
Mercury adsorption and biosensing by recombinant *E*. *coli* harboring artificial *mer* operons. After exposure to 0–400 μM Hg(II) at 37°C for 12 h, accumulated mercury and eGFP fluorescent signal of both TOP10/pPmer-LOA-Pmer-G (A) and TOP10/pPmer-HgBD-Pmer-G (C) were determined separately. The Hg(II) vs fluorescence intensity linear relationships of TOP10/pPmer-LOA-Pmer-G (B) and TOP10/pPmer-HgBD-Pmer-G (D) were in the concentration range of 6.25–200μM Hg(II). Fluorescence intensity values were normalized using the absorbance at 600 nm. The data were obtained by subtracting the value of recombinant *E*. *coli* with 0 μM Hg(II) exposure from that of each group. Data are mean ± SD from three independent assays, each from three independent cultivations.

Metal binding domains derived from MerR-like regulators were demonstrated to retain target metal ion binding capacities, and have been efficiently displayed on the microbial cell surface for heavy metal remediation [11, 16, 36, 41, 42]. Bioadsorption and biosensing of toxic mercury were previously achieved using surface display of mercury binding proteins [11, 12, 41] and whole-cell biosensing techniques [7, 9], respectively. Integration of mercury bioadsorption and biosensing in a single engineered bacterial cell was realized using an artificial double-promoter regulated *mer* operon in the study. As shown in Fig 6, two engineered bacterial cells were exposed to different concentrations of Hg(II) during the logarithmic growth period. Both the mercury binding capacities and the eGFP fluorescence of tow recombinant bacteria increased significantly with an increase of Hg(II) exposure. Bifunctional cell TOP10/pPmer-HgBD-Pmer-G was able to accumulate Hg(II) with a capacity of about 9.65 μmol/g cell at 400 μM Hg(II) exposure level, which was 5.92-fold higher than that of the control group (TOP10/pPmer-LOA-Pmer-G).

MerR and its analogues expressed in the cytosol have been demonstrated to have improved survival from Hg(II) exposed to bacterial cells [43], and to accumulate significantly more Hg(II) than cells harboring the vector alone, with no deleterious effects on cell growth [11]. In natural *mer* operon, the background expression of MerR is very low [5]. In addition, MerR represses its own expression regardless of the presence of Hg(II) [19]. As expected, TOP10 cells transformed with pPmer-LOA-Pmer-G yielded a low level of Hg(II) accumulation.

Cell surface display of MerR and its analogues were realized previously using inducible expression vectors, and exogenous inducers were added for over-expression of recombinant proteins [11, 12, 41]. Surface display of Hg(II)-binding protein was firstly induced by Hg(II) based on an artificial *mer* operon in the study. The amount of surface displayed Hg(II)-binding protein was expected to be positively correlated to the concentration of target Hg(II). The maximum binding capacity of recombinant cells with surface-exposed MerR under the control of a strong *lac* promoter was around 120 μmol/g cell [11]. Although the strength of the natural *mer* promoter is significantly weaker than the strength of the commercial inducible promoter, rational genetic designs have been proven to improve the sensitivity of the *mer* promoter [29, 44]. In order to improve the Hg(II) binding capacity, the optimized genetic elements will be used to assemble an artificial *mer* operon to improve the amount of surface displayed Hg(II)-binding protein with low concentrations of Hg(II) exposure in our future studies.

Specially, the linear relationships between Hg(II) exposure concentration and eGFP fluorescence intensity were also observed in two engineered bacteria (Fig 6B and 6D). Quantitative biodetection and bioadsorption of Hg(II) could be done simultaneously using bifunctional bacterial cells.

## Conclusions

Double-signal output mercury biosensors were developed based on both artificial dicistronic *mer* operon and artificial double-promoter *mer* operon. The whole-cell biosensors constructed in the current study tolerated high concentrations of inorganic mercury, and could detect and quantify bioavailable Hg(II) within a high concentration range. The mercury adsorptive genetic module and mercury biosensing genetic module were then integrated into an artificial double-promoter regulated *mer* operon. The bifunctional engineered cells were demonstrated to be instrumental in simultaneous detection, quantification, and capture of bioavailable Hg(II). Our findings show that it is worthwhile to develop bifunctional engineered cells based on artificial heavy metal resistance operons for simultaneous biodetection and bioadsorption toward the target metal.

## Supporting information

S1 Fig The cloning/expression region of recombinant plasmids used in this study. DNA sequence and annotation data are all marked.
(TIF)

S2 Fig The 3.7-kb *mer* operon derived from *E*. *coli* Tn21. The natural *mer* operon involves the following proteins: MerR, activator/repressor; MerT, MerP, and MerC, proteins involved in uptake of Hg(II); MerA, mercuric reductase; MerD, proposed transcriptional down-regulator.
(TIF)

S3 Fig Toxic effects of Hg(II) on the growth of three whole-cell biosensors. Exponential cultures of TOP10, TOP10/pPmer-R, TOP10/pPmer-R-G, and TOP10/pPmer-R-Pmer-G were exposed to 0, 6.25, 12.5, 25, 50, 100, 200, 400 μM Hg(II), followed by culturing at 37°C for 12 h. The absorbance of each culture was determined at 600 nm.
(TIF)

S4 Fig Time course of fluorescent signals generated by three whole-cell biosensors with 200 μM Hg(II) exposure. Exponential cultures of Top10/pPmer-R (A), Top10/pPmer-R-G (B), and Top10/pPmer-R-Pmer-G (C) were exposed to 200 μM Hg(II) at 37°C. The fluorescent signals were determined at regular time intervals. Both fluorescent signals were normalized to bacterial cell density at 600 nm. The results are shown as the mean of three independent assays ± the standard deviation.
(TIF)
