# Supplementary figures and images for: Versatile artificial mer operons in Escherichia coli towards whole cell biosensing and adsorption of mercury
Source: PLoS One. 2021 May 26;16(5):e0252190. doi: 10.1371/journal.pone.0252190 (PMC8153442; doi:10.1371/journal.pone.0252190)

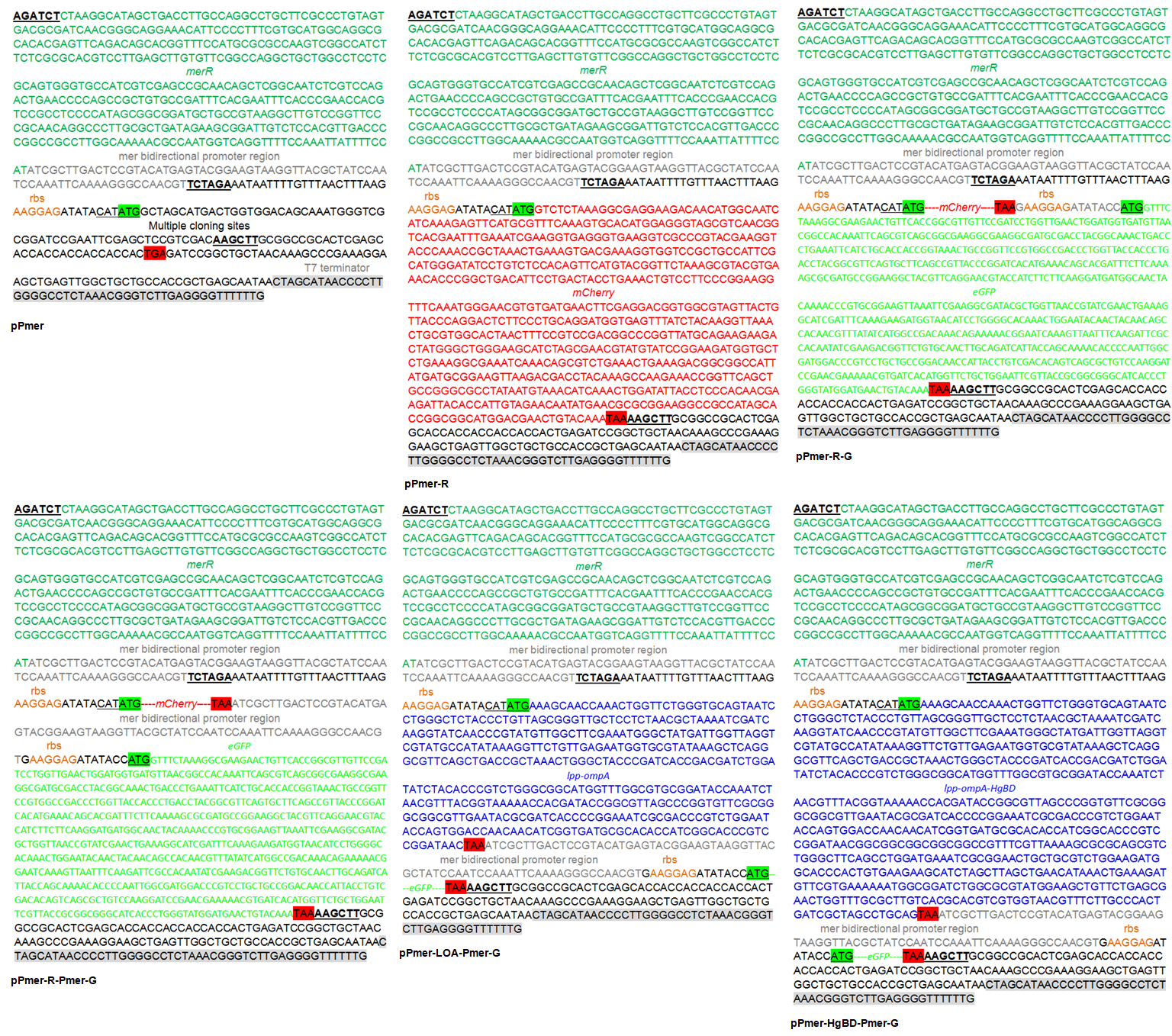

Supplement: S1 Fig — DNA sequence and annotation data are all marked. (TIF) [file pone.0252190.s001.tif]

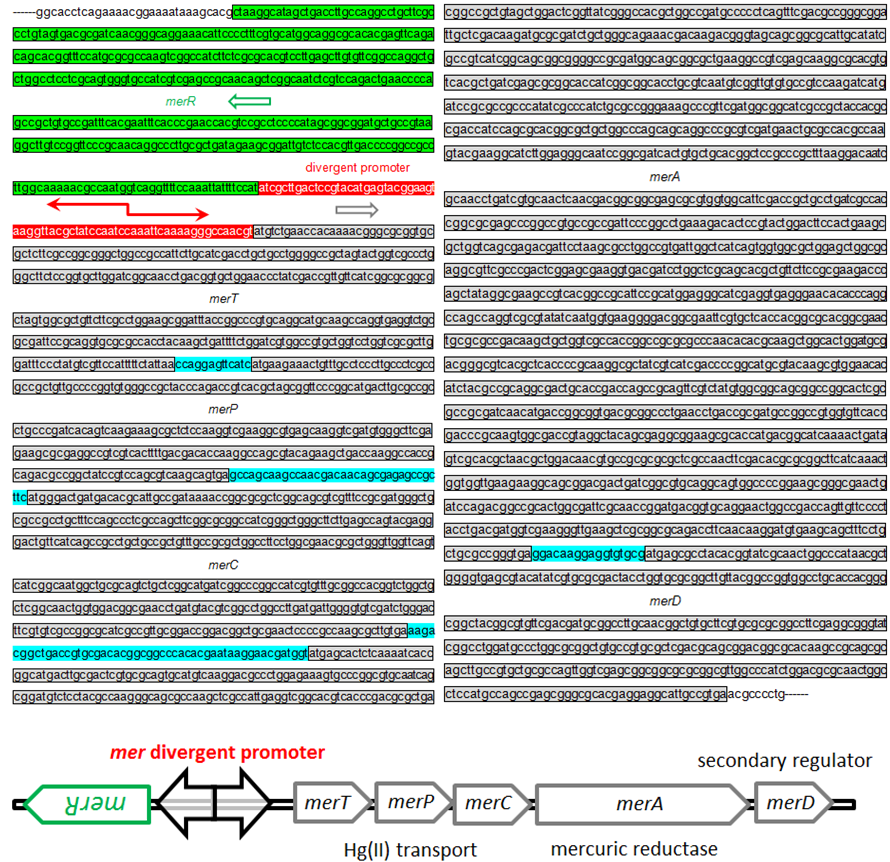

Supplement: S2 Fig — The natural mer operon involves the following proteins: MerR, activator/repressor; MerT, MerP, and MerC, proteins involved in uptake of Hg(II); MerA, mercuric reductase; MerD, proposed transcriptional down-regulator. (TIF) [file pone.0252190.s002.tif]

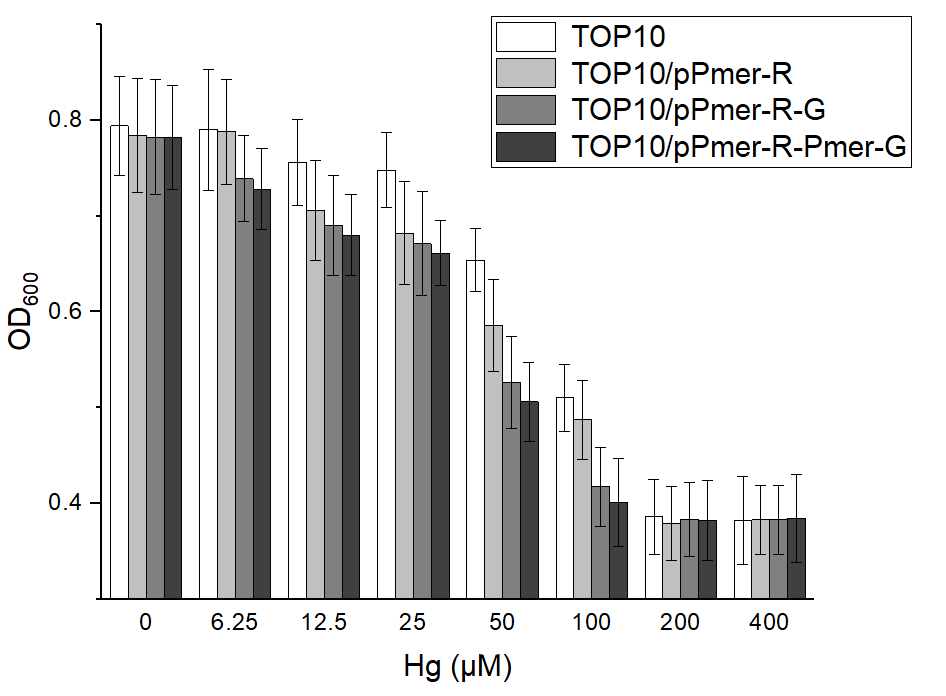

Supplement: S3 Fig — Exponential cultures of TOP10, TOP10/pPmer-R, TOP10/pPmer-R-G, and TOP10/pPmer-R-Pmer-G were exposed to 0, 6.25, 12.5, 25, 50, 100, 200, 400 μM Hg(II), followed by culturing at 37°C for 12 h. The absorbance of each culture was determined at 600 nm. (TIF) [file pone.0252190.s003.tif]

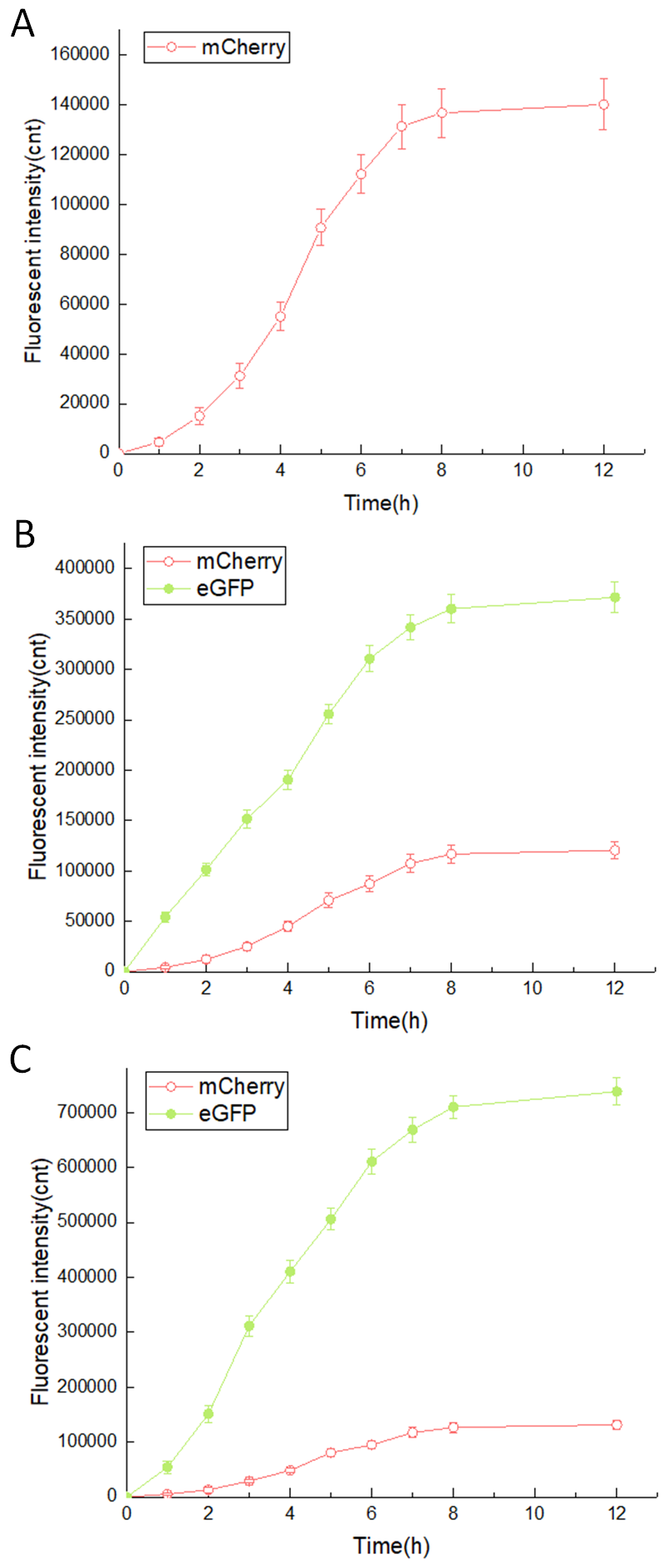

Supplement: S4 Fig — Exponential cultures of Top10/pPmer-R (A), Top10/pPmer-R-G (B), and Top10/pPmer-R-Pmer-G (C) were exposed to 200 μM Hg(II) at 37°C. The fluorescent signals were determined at regular time intervals. Both fluorescent signals were normalized to bacterial cell density at 600 nm. The results are shown as the mean of three independent assays ± the standard deviation. (TIF) [file pone.0252190.s004.tif]
